# Dental implants with versus without peri-implant bone defects treated with guided bone regeneration

**DOI:** 10.4317/jced.52292

**Published:** 2015-07-01

**Authors:** Amparo Aloy-Prósper, David Peñarrocha-Oltra, Maria Peñarrocha-Diago, Miguel Peñarrocha-Diago

**Affiliations:** 1 DDS, MSc, Collaborating Professor of the Master in Oral Surgery and Implant Dentistry, Stomatology Department, Faculty of Medicine and Dentistry, University of Valencia, Spain; 2 DDS, MSc, PhD, Junior Researcher, Collaborating Professor of the Master in Oral Surgery and Implant Dentistry, Stomatology Department, Faculty of Medicine and Dentistry, University of Valencia, Spain; 3 DDS, PhD, Full Professor of Oral Surgery, Stomatology Department, Faculty of Medicine and Dentistry, University of Valencia, Spain; 4 MD, PhD, Chairman of Oral Surgery, Stomatology Department, Faculty of Medicine and Dentistry, University of Valencia, Spain

## Abstract

**Background:**

The guided bone regeneration (GBR) technique is highly successful for the treatment of peri-implant bone defects. The aim was to determine whether or not implants associated with GBR due to peri-implant defects show the same survival and success rates as implants placed in native bone without defects.

**Material and Methods:**

Patients with a minimum of two submerged dental implants: one suffering a dehiscence or fenestration defect during placement and undergoing simultaneous guided bone regeneration (test group), versus the other entirely surrounded by bone (control group) were treated and monitored annually for three years. Complications with the healing procedure, implant survival, implant success and peri-implant marginal bone loss were assessed. Statistical analysis was performed with non-parametric tests setting an alpha value of 0.05.

**Results:**

Seventy-two patients and 326 implants were included (142 test, 184 control). One hundred and twenty-five dehiscences (average height 1.92±1.11) and 18 fenestrations (average height 3.34±2.16) were treated. At 3 years post-loading, implant survival rates were 95.7% (test) and 97.3% (control) and implant success rates were 93.6% and 96.2%, respectively. Mean marginal bone loss was 0.54 (SD 0.26 mm) for the test group and 0.43 (SD 0.22 mm) for the control group. No statistically significant differences between both groups were found.

**Conclusions:**

Within the limits of this study, implants with peri-implant defects treated with guided bone regeneration exhibited similar survival and success rates and peri-implant marginal bone loss to implants without those defects. Large-scale randomized controlled studies with longer follow-ups involving the assessment of esthetic parameters and hard and soft peri-implant tissue stability are needed.

** Key words:**Guided bone regeneration, peri-implant defects, dental implants, marginal bone level, success rate, survival rate.

## Introduction

Horizontal alveolar bone defects often result in a dehiscence or a fenestration defect exposing part of the implant surface (1,2). Several clinical studies have shown that at least 1mm of bone width buccal and lingual to the implant surface is needed to ensure long-term bone coverage and therefore implant success (3,4). When this is lacking at the moment of implant placement, guided bone regeneration has been proposed to augment the bone width in a single simultaneous surgical intervention (5,6).

An important issue is whether or not implants placed in sites associated with bone regeneration provide survival and success rates similar to those of implants placed in sites with sufficient native bone (7). Although there are a variety of case series studies on bone regeneration, three systematic reviews (1,8,9) on implant survival in sites regenerated with GBR identified only three studies (10-12) that compared implants with peri-implant defects that required bone grafts versus implants entirely surrounded by pristine bone as control implants in their analysis.

The purpose of the present study was to retrospectively evaluate whether or not implants associated with bone regeneration due to peri-implant defects show the same survival and success rates as implants placed in native bone without such defects, and to evaluate long-term outcomes of implants with dehiscences and fenestrations treated with guided bone regeneration with a minimum follow-up of three years post-loading.

## Material and Methods

-Patient selection

A retrospective controlled clinical study was made of patients with a minimum of two dental implants, one implant demonstrating a dehiscence or fenestration bony defect with exposed implant surface during implant placement and so undergoing simultaneous particulate bone grafting with resorbable membranes (test group), and the other implant site entirely surrounded by bone (control group). All implants had to be left submerged. Patients were treated between January 2005 and December 2009 at the Oral Surgery Unit of the University of Valencia in Spain and were monitored annually for a minimum of 3-years post-loading. Patients were given full information about the surgical procedures and duly signed informed consent forms. Preoperative analysis included registering complete medical histories and performing clinical and radiographic examinations. Inclusion and exclusion criteria are detailed in  Table 1. The present study is reported in accordance with the STROBE (Strengthening the Reporting of Observational Studies in Epidemiology) statement (13).

**Table 1:**
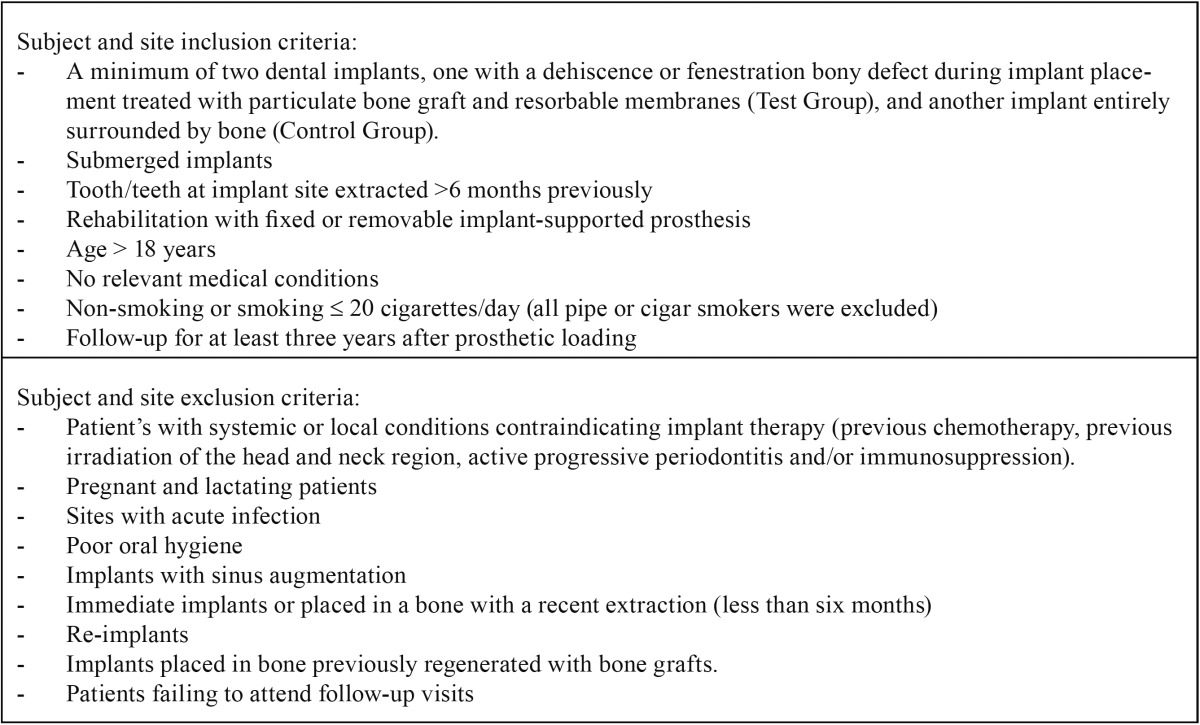
Inclusion and exclusion criteria.

-Pre-Operative Evaluation

Thorough medical histories, clinical examinations and panoramic radiographs were performed in all cases. Cone-beam computed tomographic scans were obtained to assess the availability of bone whenever the surgeon considered this necessary. Periodontal treatment was provided whenever necessary to control inflammation prior implant placement surgery. Within 10 days of the implant placement surgery, a full mouth professional prophylaxis appointment was scheduled.

-Surgical procedures

**Figure 1:**
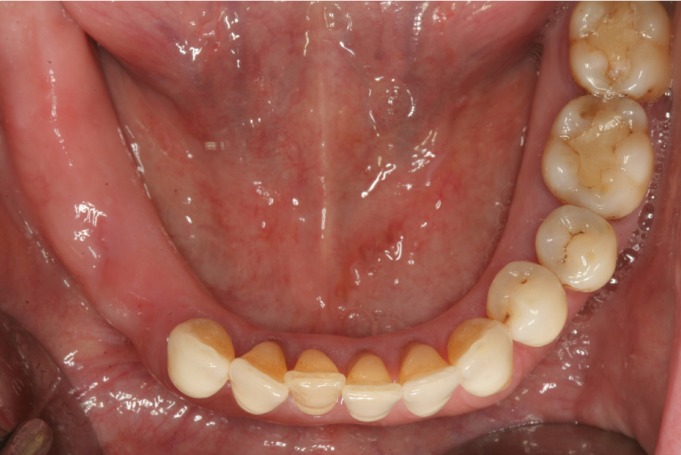
Occlusal preoperative view.

**Figure 2:**
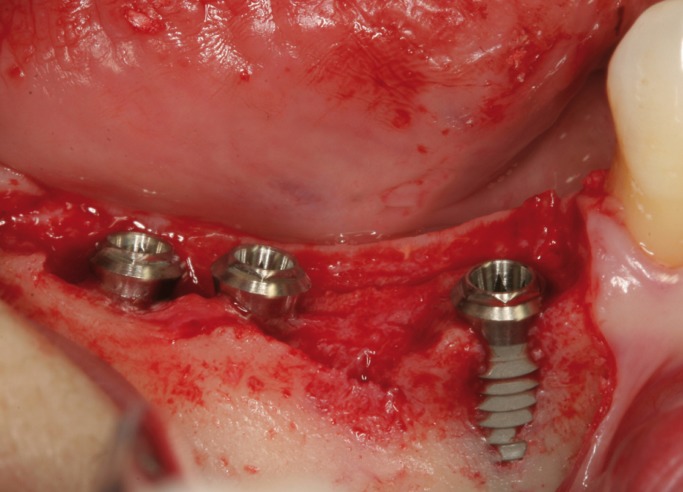
Dental implants placed in 4.5, 4.6 and 4.7 position.

**Figure 3:**
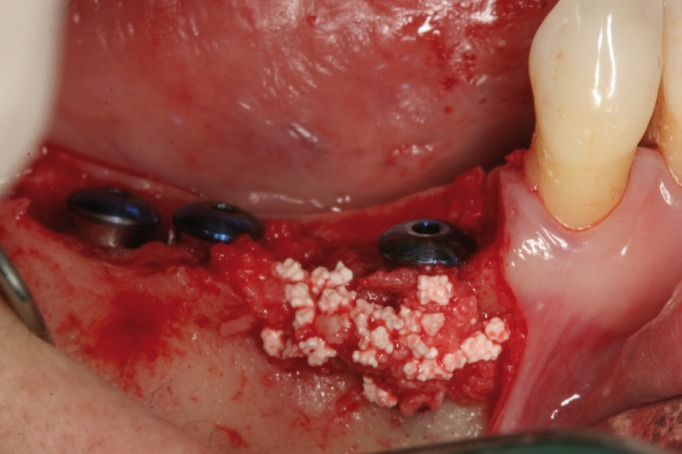
Autogenous bone graft over dehiscence.

**Figure 4:**
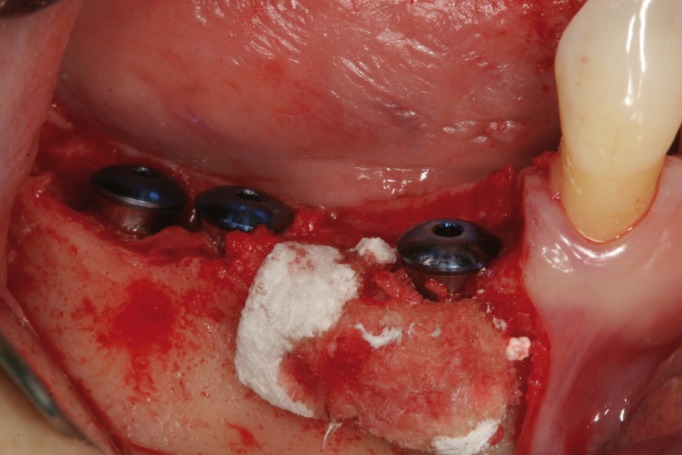
Resorbable membrane over bone graft.

**Figure 5:**
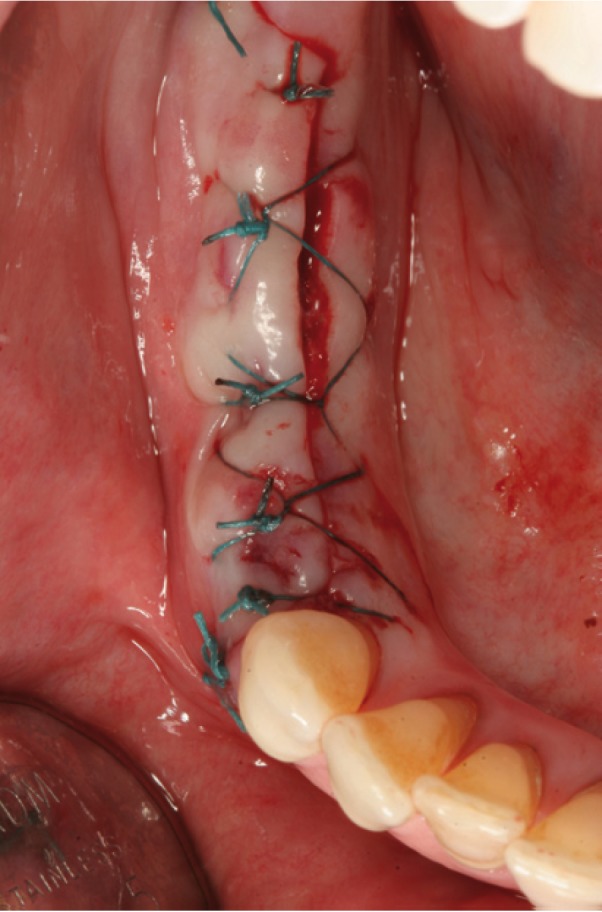
Suture.

**Figure 6:**
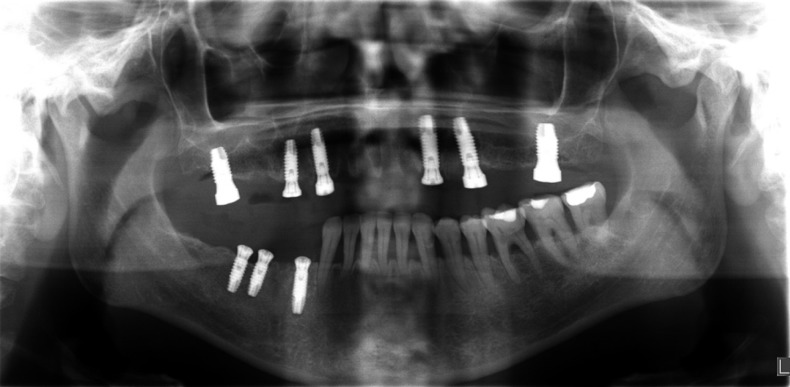
Panoramic radiography after implant placement.

**Figure 7:**
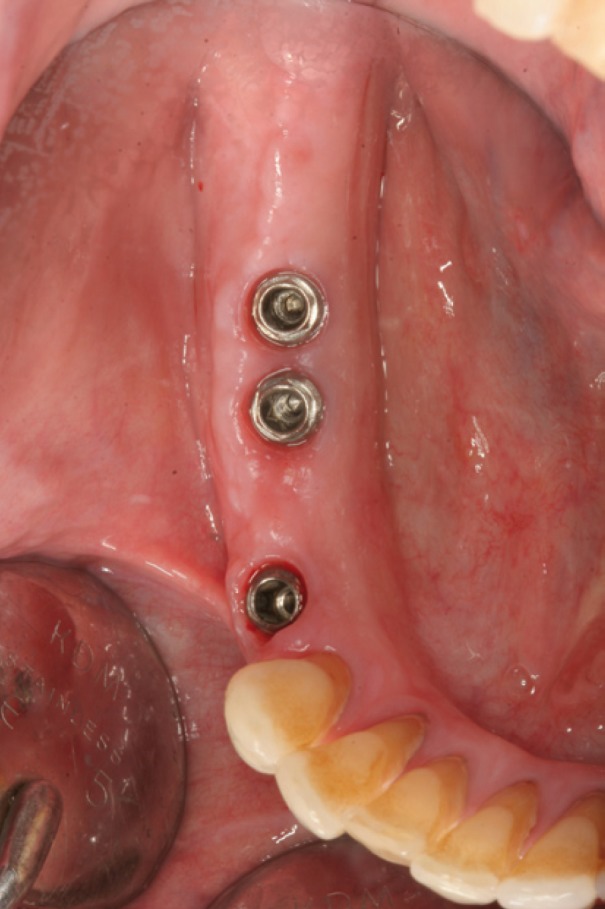
Healed soft tissues.

**Figure 8:**
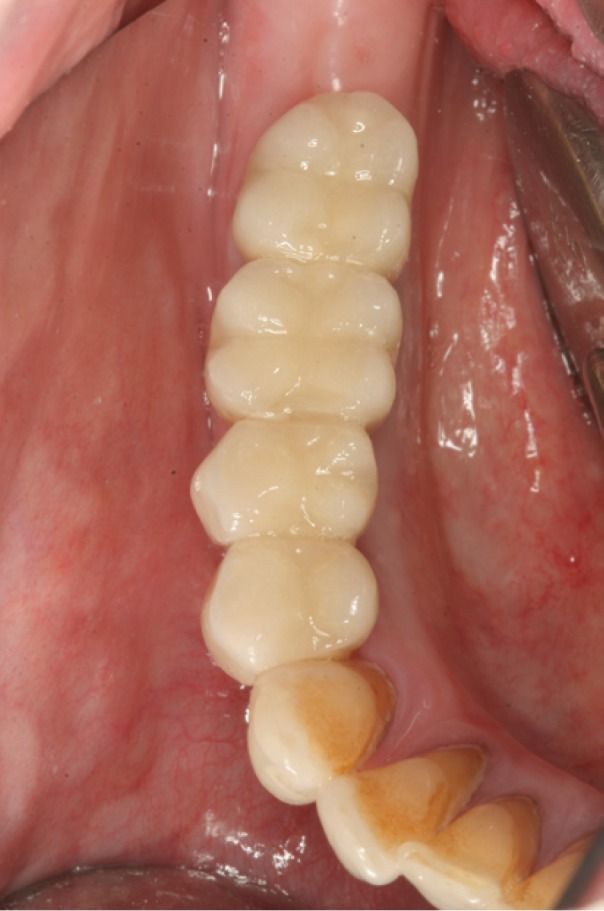
Final prosthesis placement.

**Figure 9:**
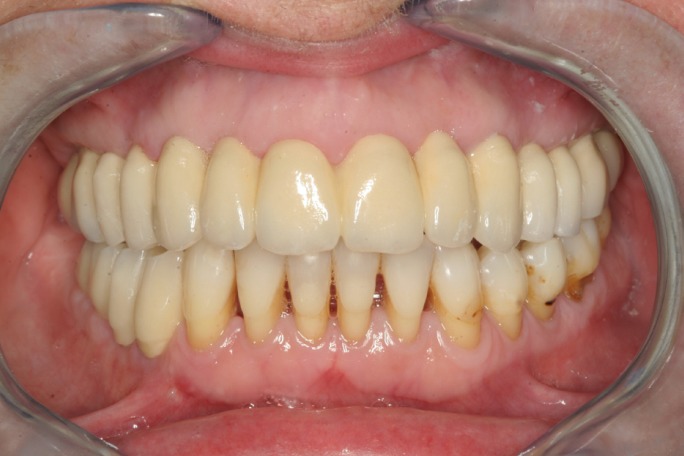
Frontal view after final prosthesis placement.

**Figure 10:**
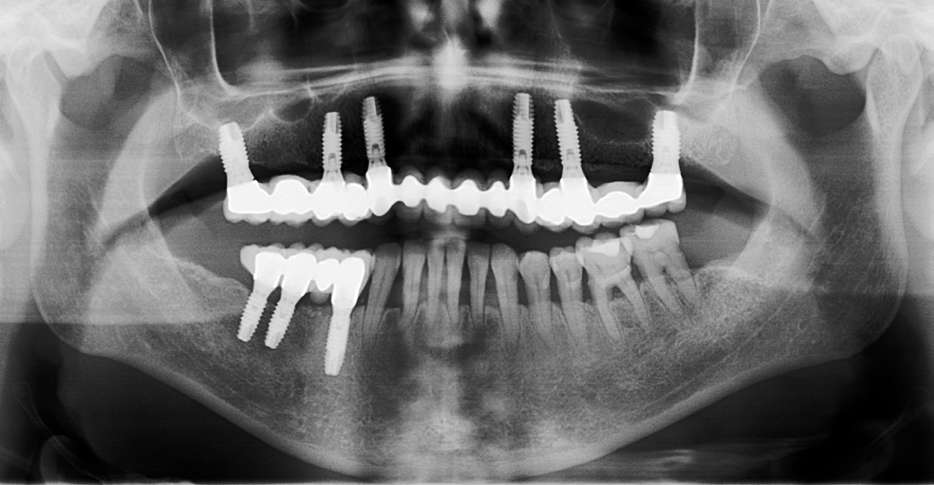
Panoramic radiography after final prostheses placement.

Surgical procedures were performed by the same surgeon with an extensive experience in regenerative procedures. All procedures were performed under local anesthesia using 4% articaine 1:100,000 adrenalin (Inibsa, Lliça de Vall, Spain) and intravenous conscious sedation with 1% propofol solution, administered by an anesthesiologist. All patients received antibiotic prophylaxis (amoxicillin and clavulanic acid, 1g every 8 hours starting 1 day preoperatively (14)). An initial incision was made slightly palatal/lingual of the alveolar crest. One or two releasing incisions were made and a mucoperiosteal flap was raised. The exposed alveolar bone was curetted to remove all soft tissues. To enhance primary stability, drills and osteotomes were combined to prepare implant beds. TSATM implants with Avantblast surface (Phibo Dental Solutions S.L., Sentmenat, Barcelona, Spain) were inserted using standard procedures following the manufacturer’s guidelines. These implants have a polished surface portion of 1.5 mm. All implants were placed with adequate primary stability (≥35 Ncm). In implants that did not need bone regeneration, bone width from the implant head to the facial plate was over 1.5 mm. In implants that needed bone regeneration, autologous bone grafts harvested from the conformation of implant beds during drilling and was adjusted to the bone contour. When the autologous bone obtained was of insufficient quantity to cover the peri-implant defects, synthetic bone (Kera-OsTM, Keramat, Coruña, Spain) was added. Grafted bone was protected with a textured collagen membrane (Lyopstic, B Braun, Aesculap, Germany). Periosteal incisions were made to allow flap mobilization and tension free primary wound closure. Implants were left submerged. Flaps were closed with horizontal sutures using Polisoft® 4/0 sutures (Sweden & Martina, Due Carrare, Italy) (Figs. 1-10). Patients were prescribed amoxicillin and clavulanic acid 1g (GlaxoSmithKline, Madrid, Spain) twice daily for six days, 600 mg ibuprofen (Bexistar, Laboratorio Bacino, Barcelona, Spain) three times per day for five days and mouthrinse with chlorhexidine 0.12% (GUM, John O Butler/Sunstar, Chicago, IL, U.S.A.) twice daily, commencing three days prior to surgery and for two weeks thereafter. Patients were instructed in adequate hygiene maintenance and a soft diet was recommended for eight weeks. Patients were not allowed to use removable prostheses for three weeks after bone grafting surgeries. A soft diet was recommended for one week and patients were instructed to avoid brushing or any other trauma to the surgical sites. Sutures were removed two weeks after surgery.

Second surgeries were performed two or three months after implant placement and final fixed prostheses were placed one month later.

-Data collection and follow-up

All data collection was carried out by a single trained clinician, different from the surgeons or the prosthodontist, following a pre-established protocol. All patients were included in a maintenance program involving annual examinations and professional prophylaxis.

Patient age (at implant placement), gender, hygiene and smoking habits (none / <10 cigarettes per day / 10-20 cigarettes per day) were registered. For each implant, the position, and the type and dimensions of defects (dehiscence/fenestration) were registered. Defects were measured using a millimetric periodontal probe (Hu-Friedy UNC, Chicago, IL, USA) placed parallel or perpendicular to the long axis of the implant. Measurements were recorded to the nearest 1mm mark. The use of temporary prostheses (yes or no), definitive prosthesis design (single, partial or complete – fixed or overdenture) and type of prosthesis (cemented or screwed) were recorded.

All patients were included in a maintenance program involving annual examinations and occlusal adjustment was performed when necessary.

The following outcome measures were recorded:

Receptor site healing: Wound dehiscence with bone graft exposure.

Implant survival: The criteria for implant failure were implant mobility or the removal of stable implants due to progressive peri-implant marginal bone loss or infection.

Implant success: The definition of implant success was based on the clinical and radiographic criteria put forward by Albrektsson *et al.* (15).

Radiographic peri-implant marginal bone loss: Intraoral radiographs were made at the moment of prosthetic loading (baseline), one-year post-loading and at 3 year control radiograph, using the X-MindTM intraoral system (Satelec-Pierre Rolland Group, Merignac, France) and an RVGTM intraoral digital receptor (Dürr Dental, Bietigheim-Bissingen, Germany) with the aid of a Rinn XCPTM (Dentsply Rinn, Elgin, IL, U.S.A.) to achieve parallelism. Evaluation of the marginal bone level around implants was performed using image analysis software (Autocad 2006, version Z 54.10, Autodesk, USA), which is designed to compensate for radiographic distortion. Each image was calibrated using the known length of the implants. The vertical distance from the outer edge of the implant shoulder (reference point) to the most coronal point of bone-to-implant contact was evaluated at the mesial and distal aspect of each implant. Peri-implant marginal bone resorption at 3-year post-loading was calculated from the change in bone level between the 1-year post-loading and the 3-year control radiograph; for each pair of measurements (mesial and distal) the largest value was used. Intra-examiner calibration was analyzed before evaluating the entire implant sample by reassessing bone loss at a total of 30 randomly selected sites (using the random function of Microsoft Excel 2010) on duplicate measurements performed on different days. An intraclass correlation coefficient of 0.833 was obtained, showing a high concordance between the two sets of data. According to Dahlberg’s d-value, a 0.046 mm error was estimated for the measurement method.

-Statistical Analysis 

Statistical analysis was performed using non-parametric tests for implant success, as this was a non-continuous variable, and marginal bone loss, as this was an asymmetric distribution. The Chi2 test and the Mann-Whitney test (MW) were used to evaluate homogeneity within the two groups in relation to the demographic factors, clinical parameters, implant survival and success rates. The MW test was used to compare bone loss between groups. The relationship between the implant failure and bone loss with respect to age, gender, smoking and hygiene habit, position and location of the implants, type defect and defect dimensions or type of graft was studied with non-parametric tests. The statistical power for this test was 99.5% to detect an effect of 0.27 with a confidence of 95% and alpha set at 0.05. Statistical analysis was performed using SPSS 17.0 software (SPSS Inc. Chicago, IL, U.S.A.).

## Results

A total of 129 patients with submerged dental implants placed with dehiscences and fenestrations and treated with particulate bone graft and implants without these bony defects in the same patient were included. Eleven patients were excluded as a result of failing to attend the scheduled recall visits and 20 because control implants were not submerged.

The final study sample included 72 patients (37 women and 35 men) with a mean age of 55.4 ± 11.7 (25-87). Hygiene maintenance was good in 47 patients and regular in 25. Forty-eight patients were non-smokers, 11 smoked less than 10 cigarettes per day, 7 between 10 and 20 cigarettes and 6 were ex-smokers.

A total of 326 dental implants (142 test group, 184 control group) were placed. Out of 183 implants in test group, 162 had dehiscences and 21 with fenestrations. The mean dimensions of the resulting dehiscence defects were: 1.92±1.11 mm (range 1-6) length and 3.29 mm (range 3 to 5.5 mm) width. The mean dimensions of the resulting fenestration defects were: 3.34±2.16 mm (range 1-8) and 2.1 mm (range 1.5 to 3 mm) width. The distribution of patient or implant variables is described in  Table 2. No statistically significant differences were found between the two study groups regarding implant variables.

**Table 2:**
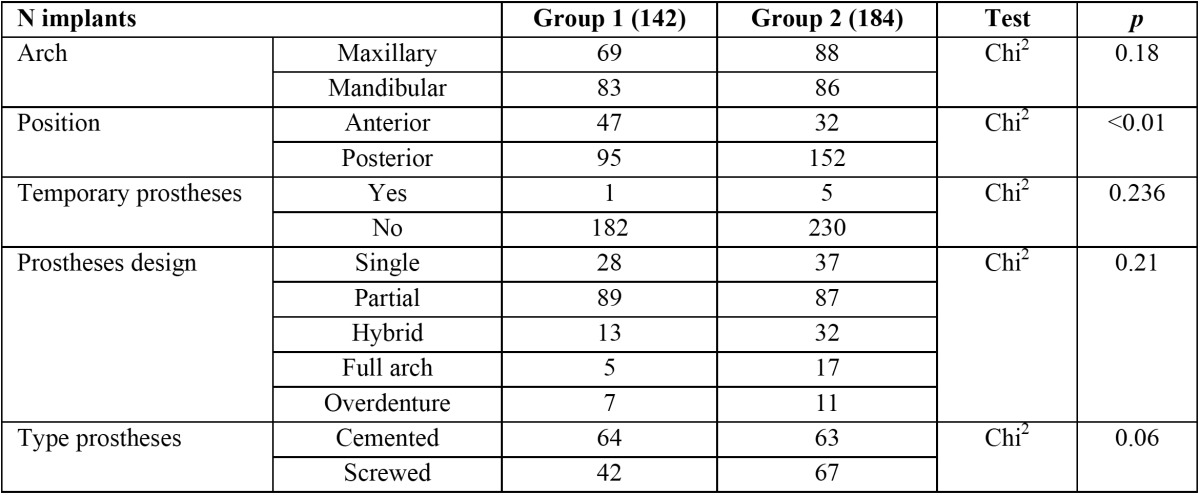
Description of implants distribution sample.

-Receptor site healing

Wound dehiscence with membrane exposure during the early postoperative period occurred in twelve grafted sites in twelve pa-tients (8.4% membrane exposure rate). These exposures did not exceed 3mm in diameter. In these cases, 0.2% chlorhexidine gel was prescribed three times daily over the exposed membrane for 6 weeks after surgery. All sites re-epithelialized uneventfully.

-Implant survival, success rates, and peri-implant marginal bone loss

Seven implants in seven patients in the test group were lost, all of them before loading. Five implants in four patients in the control group failed to osseointegrate and were removed, four before loading and one after loading (at eight months after loading). The distribution of implant failures is described in  Table 3.

**Table 3:**
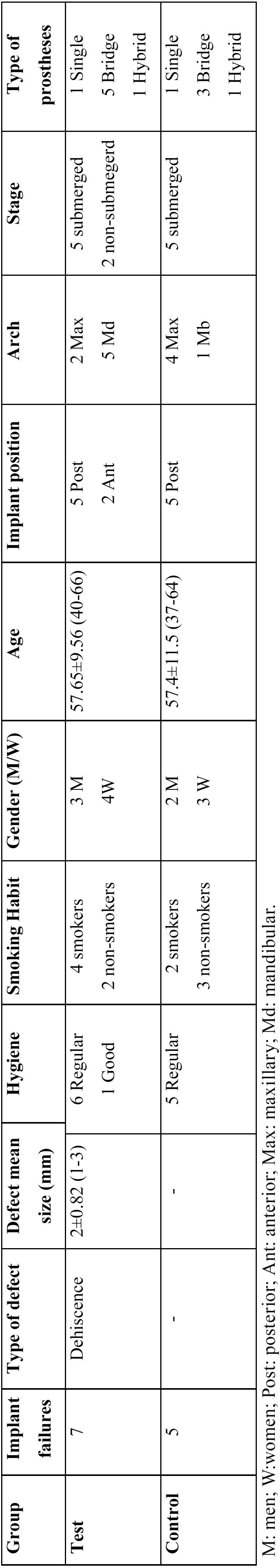
Description of implant failures.

Implant survival rates were 95.7% for the test group and 97.3% for the control group; the difference was not statistically significant (p-value = 0.542). Mean peri-implant marginal bone loss one year after 1-year loading was 0.44 mm (range 0.1-1.7, SD 0.27 mm) for the test group and 0.39 mm (range 0.1-1.3, SD 0.23 mm) for the control group. Bone loss was slightly higher for implants placed in grafted bone, although differences were not statistically significant (*p*=0.213).

At 3 years post-loading, no further implants were lost. 93.6% of the implants in the test group and 96.2% in the control group showed stable alveolar bone levels; no statistically significant differences were found (*p*=0.587). Mean peri-implant marginal bone loss at 3 years post-loading was 0.54 mm (range 0.1-2.1, SD 0.26 mm) for the test group and 0.43 mm (range 0.1-1.7, SD 0.22 mm) for the control group. Differences between both groups were not statistically significant (*p*=0.893).

No significant differences were found between implant failures/bone loss and patient factors (age, gender, smoking habit) or implant variables (location, type defect, defect dimensions, prostheses). However, implant failures were more frequent among patients with a regular oral hygiene (91.6%) than good (8.4%); these results showed a moderate tendency to significance but were nevertheless non-significant (*p*-value 0.06).

## Discussion

Peri-implant bone defects occurring during the surgical implant placement procedure are a frequent problem in daily practice, one with considerable clinical relevance. For this reason, the present study set out to evaluate the long-term outcome of implants with dehiscences and fenestrations treated with guided bone regeneration with a minimum follow-up of three years. The study analyzed complications associated with guided bone regeneration, implant survival and success rates and peri-implant marginal bone loss.

Soft tissue thickness and primary flap closure are important aspects of surgery for the maintenance of wound stability during healing (16). Early exposure of the membrane, with consequential bacterial contamination of the healing tissues, hinders bone regeneration, despite careful remedy with chlorhexidine applications (17,18). Nowzari and Slots (19) showed that implant sites with submerged barrier membranes remained free of cultivable microorganisms throughout their nine-month study and experienced significantly more osseous healing than sites with prematurely exposed membranes. The application of chlorhexidine has been recommended for cases of membrane exposure (2,3). In the present study, wound dehiscence with membrane exposure occurred at twelve grafted sites in twelve patients during the early postoperative period.

There is insufficient evidence in the literature in support of the need to cover the dehiscence and fenestrations for ensuring long-term implant survival (20). To address this issue, randomized clinical trials that compare treatment with GBR vs. non-treatment of fenestrations/dehiscences should be performed. But to date, no research has been published with these precise characteristics. Dahlin *et al.* (21) conducted the first split-mouth study on implants (machined titanium) with fenestrations treated with or without e-PTFE membrane; there was a significant bone gain in the group treated with GBR in comparison with the non-treated group, but there was no difference in the implant survival rates between groups. The authors showed that membranes alone, without the additional aid and sustenance provided by graft materials, will allow the formation of new bone; however, it could not be demonstrated that the realization of this bone augmentation was necessary for implant survival. The present article was only able to demonstrate that dehisced implants, if properly treated with grafting materials and membranes, may lead to a survival rate of implants similar to that obtained in implants completely embedded in native bone, but it was not designed to demonstrate whether GBR is really needed in case of fenestrations/dehiscences.

Only four studies have set out to determine whether the survival of implants with bone defects treated with peri-implant regenerative procedures is comparable to implants without defects in the same patient (7,10-12). According to these studies, survival and success rates were not significantly different between implants in regenerated and non-regenerated bone. In the present study, implant survival rates were 95.7% (test) and 97.3% (control) and implant success rates were 93.6% and 96.2%, respectively, at 3 years post-loading. These results compare well with systematic reviews reporting survival rates of implants placed into sites with regenerated bone ranging from 79% to 100%, with the majority of studies indicating >90% after at least 1 year of function (9,22). The survival rates obtained in these systematic reviews were similar to those reported in the meta-analysis of prospective longitudinal studies for implant therapy with and without bone regeneration after at least 5 years (23).

The assessment of bone levels around implants with buccal or lingual defects presents a methodological challenge. Schliephake *et al.* (24) showed that determining peri-implant bone levels from periapical radiographs, reformatted CT scans or direct magnification imaging does not provide valid data on the circumferential bone level in implants with buccal bone defects, but does generally reflect the bone level on the lingual side. Periapical radiographs were found to obtain the best correlation, though they tend to overestimate the bone anchorage of these implants. Authors such as Mayfield *et al.* (10) have hypothesized that distal and mesial marginal bone values reflect the buccal bone margin. Jung *et al.* (5) found minimal mean changes (0.03-0.13mm) of the marginal bone level at test sites and control sites between baseline, 3- and 5-year examinations. In a retrospective multi-center study, the mean radiographic bone loss over a 74-month period of implants loaded in regenerated bone was 0.64mm (0.3-0.8mm) (25). A further mean annual crestal bone loss ranging from 0.05-0.13mm after the first year of function (i.e. after abutment connection) has been reported (26-27). In this study, mean peri-implant marginal bone loss after 3-years post-loading was 0.54 ± 0.26 mm for the test group and 0.43 ± 0.22 mm for the control group, without significant differences between groups. These results demonstrated that bone regenerated by GBR in peri-implant bone defects remains as stable over time as pristine periimplant bone. These data compare well with results reported in previous studies including implants in pristine as well as regenerated bone (10,12).

In the present study, re-entries were not performed, so it provides no evidence on the effectiveness of this technique on bone regeneration. In addition, it should be noted that all procedures were performed by the same oral surgeon with extensive clinical experience in regenerative procedures, which may limit the extrapolation of the results. Nevertheless, the lack of appropriate controls and the lack of good outcome measurements that assess aesthetic demands preclude definitive conclusions. Further long-term studies with appropriate controls and larger sample sizes with longer follow-ups should be conducted in order to confirm or reject these findings.

## Conclusions

Within the limits of this study, implants with peri-implant defects treated with guided bone regeneration exhibited similar survival and success rates, and peri-implant marginal bone loss to implants without these defects. Implant survival and success rates were high in both groups; marginal bone levels were within the range of published data.
